# Unsupervised recognition of components from the interaction of BSA with Fe cluster in different conditions utilizing 2D fluorescence spectroscopy

**DOI:** 10.1038/s41598-022-20768-6

**Published:** 2022-10-07

**Authors:** Mohsen Kompany-Zareh, Somayyeh Akbarian, Mohammad Mahdi Najafpour

**Affiliations:** 1 Department of Chemistry, Institute for Advanced Studies in Basic Sciences (IASBS), Zanjan, 45137-66731 Iran; 2 Center of Climate Change and Global Warming, Institute for Advanced Studies in Basic Sciences (IASBS), Zanjan, 45137-66731 Iran; 3 Department of Chemistry, Dalhousie University, P.O. Box 15000, Halifax, NS B3H 4R2 Canada; 4 Research Center for Basic Sciences and Modern Technologies (RBST), Institute for Advanced Studies in Basic Sciences (IASBS), Zanjan, 45137-66731 Iran

**Keywords:** Fluorescent probes, Bioinorganic chemistry

## Abstract

The excitation-emission fluorescence spectroscopy combined with three-way analysis was applied for discriminating the pure BSA and BSA/Fe_3_O(OAc)_6_ClO_4_ (Fe) using unsupervised classification methods. Herein, the interaction of bovine serum albumin (BSA) and Fe clusters as an artificial enzyme is studied by extracting the intrinsic excitation-emission (EEM) fluorescence of BSA. The conformation of BSA changes with pH, temperature, and Fe concentration. Three-way fluorescence data were recorded for BSA and BSA/Fe during different days. The obtained results showed that the Fe clusters cause changes in the structure of BSA conformation as a function of pH, temperature, and Fe concentration. Also, the denaturation pathway of the BSA molecule is significantly different in the presence of Fe clusters. Both techniques of PARAFAC and PCA were used in the excitation-emission fluorescence matrices (EEM) of solutions at three different pH (5.0, 7.0, and 9.0) and temperatures (15.0, 25.0, and 35.0 °C) values. Also, we reported the results of the change in concentrations of Fe (4.0, 6.0, and 8.0 mg) using these methods. These three amino acids (tyrosine, tryptophan, and phenylalanine) indicate all datasets and their similarities and differences. The spectral differences were more remarkable in different pH values compared to different temperatures. Also, we could distinguish between the groups of protein samples properly in different concentrations of Fe using low-cost EEM spectral images and PARAFAC.

## Introduction

Studying protein conformational changes is essential for catalysis, regulation, and substrate recognition^1,2^. Investigating the correlation between the structure and function of proteins and monitoring different levels of changes in their structures have made a significant area in biochemical and biophysical research that may result in a remarkable change in protein function^3,4^. One of the conceivable methods for simulating the PSII is using bovine serum albumin (BSA) as an emulator for stabilizing proteins and Fe_3_O(OAc)_6_ClO_4_ cluster in the role of Mn cluster to make BSA with Fe cluster (BSA/Fe). BSA is a globular protein with a molecular weight of about 66 kDa. It is used in many biochemical applications because of its stability and binding ability to water, salts, and fatty acids^5^. The adsorption of various materials on proteins and their interactions has been widely studied^6^. It has been shown that electrostatic, hydrophobic, and specific chemical interactions play essential roles^7^. Conformational stability of BSA during different days is considered, and the effect of the Fe cluster on the stability is investigated. The question is to find the effectiveness of the Fe cluster on the BSA spectrum and the role that included Fe concentration play. More recently, interaction and combination of many different materials, including nanoparticles, with proteins such as BSA have been studied using various techniques such as Fourier transform infrared (FTIR) and circular dichroism (CD) spectroscopy, Isothermal Titration Calorimetry (ITC), Surface Plasmon Resonance (SPR), mass spectrometry, and fluorescence spectroscopy^8–11^. Among these techniques, a non-destructive method with high selectivity, speed, and relatively low-cost is fluorescence spectroscopy^12–15^ and the obtained signal is 1000 times more sensitive than absorption-based spectrophotometric techniques^16,17^. Therefore, this technique is an advantageous tool for studying protein structure, function, and interaction between them^18–20^.

The fluorescence characterization of BSA is due to tryptophan (Trp) and tyrosine (Tyr) residues with different excitation and emission wavelengths. The BSA conformation is highly influenced by pH, temperature change^21^, and other contributions of clusters that were adsorbed on the proteins. Also, the local environment changes of the amino acids are because of BSA conformational changes such as binding to substrates, and denaturation, which affect their fluorescence. In this way, to survey the interaction of BSA with the Fe cluster, the fluorescence excitation-emission matrix (EEM) is considered in this study. We expect that the interaction of different contributions of the Fe cluster is effective in the extent of conformational changes in BSA.

Excitation-emission matrix (EEM) fluorescence spectroscopy as a multidimensional technique^22^ gives more information than single wavelength fluorescence^23–25^, and it is highly selective, sensitive, and low-cost^22^ EEM fluorescence bands are usually overlapped. Multiway techniques can extract components information from the stacked fluorescence data cube of fluorophore mixture samples. Exceptionally multivariate techniques for complicated biological samples benefit from the simultaneous resolution of contributions from the different fluorescent species. Multiway techniques can be applied for data exploration without using any previous information about data. Also, they are mainly used for classification, to group samples with similar characteristics in the unknown set of samples, or to characterize different properties of unknown samples quantitatively as a calibration modeling. Unsupervised multivariate classification methods are called pattern recognition^26^, such as principal component analysis (PCA), hierarchical cluster analysis (HCA), and parallel factor analysis (PARAFAC) methods^12,27^. Unsupervised is a type of classification in which there is no information about the classes. The separation of classes is based on the data pattern using PCA and PARAFAC as the component models.

In the present study, the applied pattern recognition techniques are PCA and PARAFAC. PCA is usually employed to explore data to evaluate the importance of different dimensions, cluster and classify samples, and distinguish potential outliers. PARAFAC is a multiway method used to trilinearly resolve the data and mathematically separate the profiles of fluorescence species, even in the presence of high overlap^28^. An advantage of PARAFAC to PCA is resolving the data cube into three series of profiles using a simple alternating least squares algorithm, which is assisted with constraints^29,30^. Therefore, the PARAFAC model can describe the contribution of each fluorescent species in three-way data, showing their relative concentrations and pure spectral profiles in the analyzed samples^17^. The advantage of PCA is simplicity and a more flexible model compared to PARAFAC and orthogonal profiles. One of the goals of this study is to compare PARAFAC to PCA to see which methods show higher performance in clustering the fluorescence data from different samples.

We considered this study's two sample sets of BSA and BSA/Fe. The bovine serum albumin/Fe was synthesized based on the below instructions. Fluorescence spectra were repeatedly measured at pHs = 5.0, 7.0, and 9.0 and temperatures of 15.0, 25.0, and 35.0 °C for both BSA and BSA/Fe samples. It was interesting to find which experimental condition was more effective on the spectral behavior of BSA and BSA/Fe and which one of the species was more affected. Samples with different known contributions to the Fe cluster were measured on other days. Then, the obtained EEM fluorescence data sets were resolved using principal component analysis (PCA) and parallel factor analysis (PARAFAC). The resolved profiles from both techniques were utilized for unsupervised recognition of the members of different classes of protein samples in other conditions. Resolution methods were exerted to resolve the data into profiles to interpret observed evolution in data arrays. Then, the effects of pH, temperature, and the contribution of Fe changes on the BSA conformation were investigated by following the Trp and Tyr EEM fluorescence changes in the presence and absence and presence of different amounts of Fe cluster.

## Results and discussion

The alteration in shape, usually the tertiary structure, of a protein is a result of changes in the environmental conditions (pH, temperature, and ionic strength), binding of a ligand (receptor), or binding of substrate (enzyme). The structure and shape of proteins can influence their functions, such as bioavailability and catalytic activity. Therefore, investigation of protein structure is essential^31^. Structure change of proteins results in variation in their spectral behavior. In this way, spectroscopic techniques are a rapid and low-cost approach for studying protein conformation and structure changes. One of the influential factors on the BSA confirmation at the tertiary structure level by creating different protonated and deprotonated states of amino acids in proteins is pH altering in the solution. As a result, the changes occur in their functional groups and local charges in internal and surface amino acids^13^. Therefore, the net charge of BSA is very sensitive to the pH of the solution so that with pI indicating the isoelectric point (for BSA is 4.7), positive charge for pH < pI, neutral for pH = pI, and negative charge for pH > pI^21^. In addition, change in location of different amino acids as the result of variation in protein conformation that is a function of alteration in protein environment may be detected via following the fluorescence changes, which was performed for BSA protein or BSA/Fe complex with the change of pH. The three amino acids, including tryptophan (Trp213 and Trp134 with 91% contribution), tyrosine (Tyr), and phenylalanine (Phe), are responsible for creating BSA fluorescence^32,33^. Also, the thermal denaturation of the proteins for understanding protein stability is a complex process and is considered in some reports^21^. By increasing the temperature, the native form of the protein is unfolded, and free SH groups of the hydrophobic regions can participate in new intermolecular interactions^34^. A cluster inside the protein, such as the Fe cluster, also affects their conformation and spectral behavior as a function of condition variations^13^.

The synthesized samples were characterized. XRD patterns were investigated for the Fe cluster, BSA, and BSA/Fe cluster in Fig. S1. Although Fig. S1 showed the corresponding patterns for Iron(III) oxo acetate perchlorate hydrate, BSA and BSA/Fe clusters showed only a broad peak at 2θ = 13°–30°, which corresponded to the amorphous structure. We suggested that Fe_3_O (OAc)_6_(H_2_O)_3_^+^ units significantly separated each other and dispersed into the BSA structure. Thus the corresponded patterns of the Fe cluster were not observed in BSA/Fe cluster.

Also, SEM images from the BSA/Fe cluster surface show a worm-like structure on the surface of this compound (thickness: 5–10 nm; length: 50–100 nm) (Fig. S2a,b). EDX-SEM showed that iron is dispersed on the surface of BSA, and no iron-oxide particles were observed on the surface of BSA.

## BSA samples

### Effect of pH

**Figure 1:**
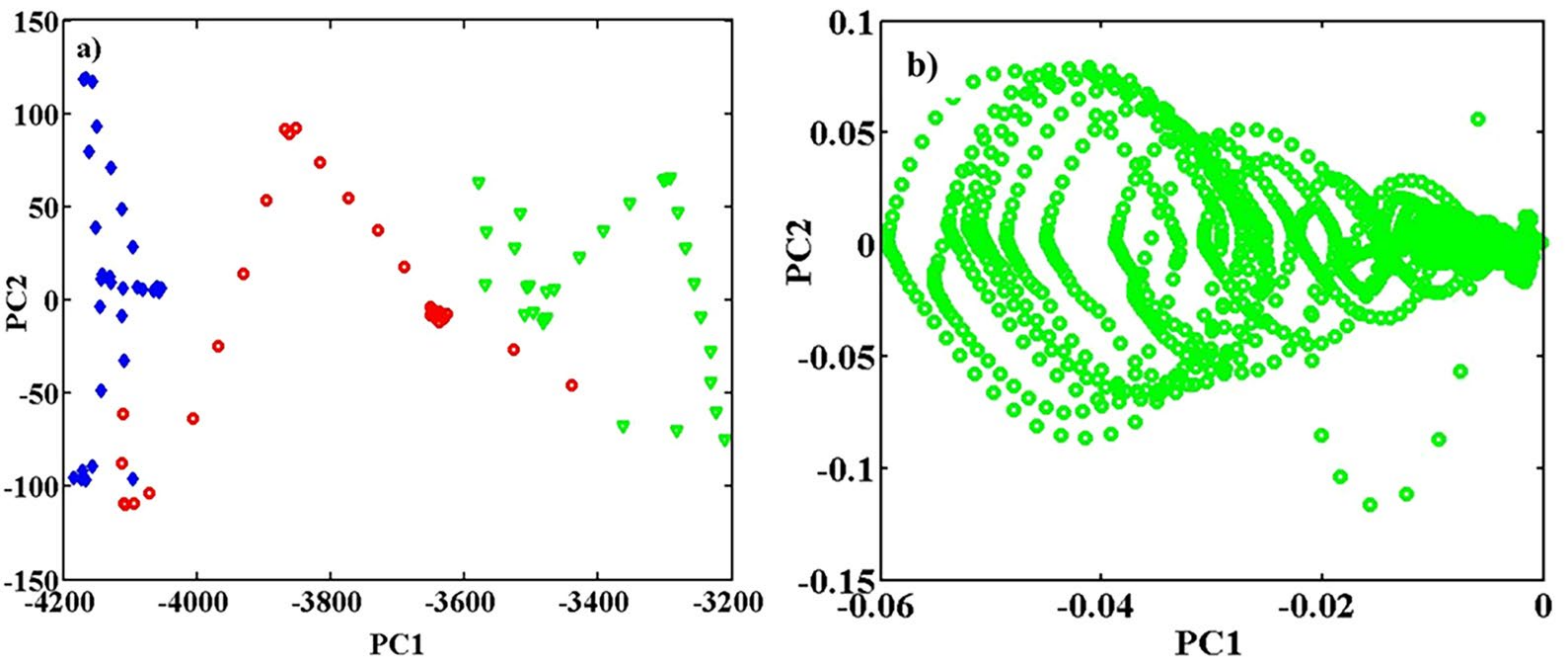
PCA with two PCs, performed on unfolded the augmented array with the size 90 (sample) × 1891 (λ_ex_ × λ_em_). Scattered score from the sample mode is plotted as (**a**) PC2 vs. PC1. Scattered loading in the wavelengths mode is illustrated as PC2 vs. PC1, (**b**). In parts (**a**), the blue lozenges are related to pure BSA at pH = 5.0, the red circles at pH = 7.0, and the green triangles at pH = 9.0.

The effect of change in pH of protein solution, from acidic to neutral and basic conditions, on fluorescence of pure BSA, using replications of the spectra, for classification purposes is surveyed below. At first, the fluorescence spectra of samples were recorded at pH = 5.0, 7.0, and 9.0 with 30 sampling repetitions for any class of sample on three different days. Before analysis, the Rayleigh scattering and extra areas were removed from EEM fluorescence images. Therefore, sample matrices of pure BSA at pH = 5.0, 7.0, and 9.0 were row-wise augmented. As a result, an array with of size of 31 (λ_ex_) × 61 (λ_em_) × 90 (sample) was obtained, and the sample replicates were analyzed. Therefore, more reliable information was extracted^35^. The array obtained from the augmented data with size 31 (λ_ex_) × 61 (λ_em_) × 90 (sample) clarified the effect of pH on pure BSA spectra. As shown in Fig. 1a clearly, the distribution and position of points for pure BSA are different at different pH values.

*PCA* At first, PCA with two PCs was performed on the augmented metricized data from three classes with dimensions of 90 (sample) × 1891 (λ_ex_ × λ_em_). A scatter plot of scores from the mode of samples is shown in Fig. 1a (PC2 vs. PC1), and the loadings from the mode of wavelengths are shown in Fig. 1b (PC2 vs. PC1).

In Fig. 1a, the pure BSA samples are successively arranged, however not completely separated, at pH = 5.0, 7.0, and 9.0. PCA showed that PC1 has the main role in discrimination between samples in mentioned pHs. Plot (b) in Fig. 1 is the loadings scatter plot, which shows the effective excitation and emission wavelengths for discriminating the pure samples in different pHs. A three-way analysis was applied using the PARAFAC method to confirm discrimination between samples.

**Figure 2:**
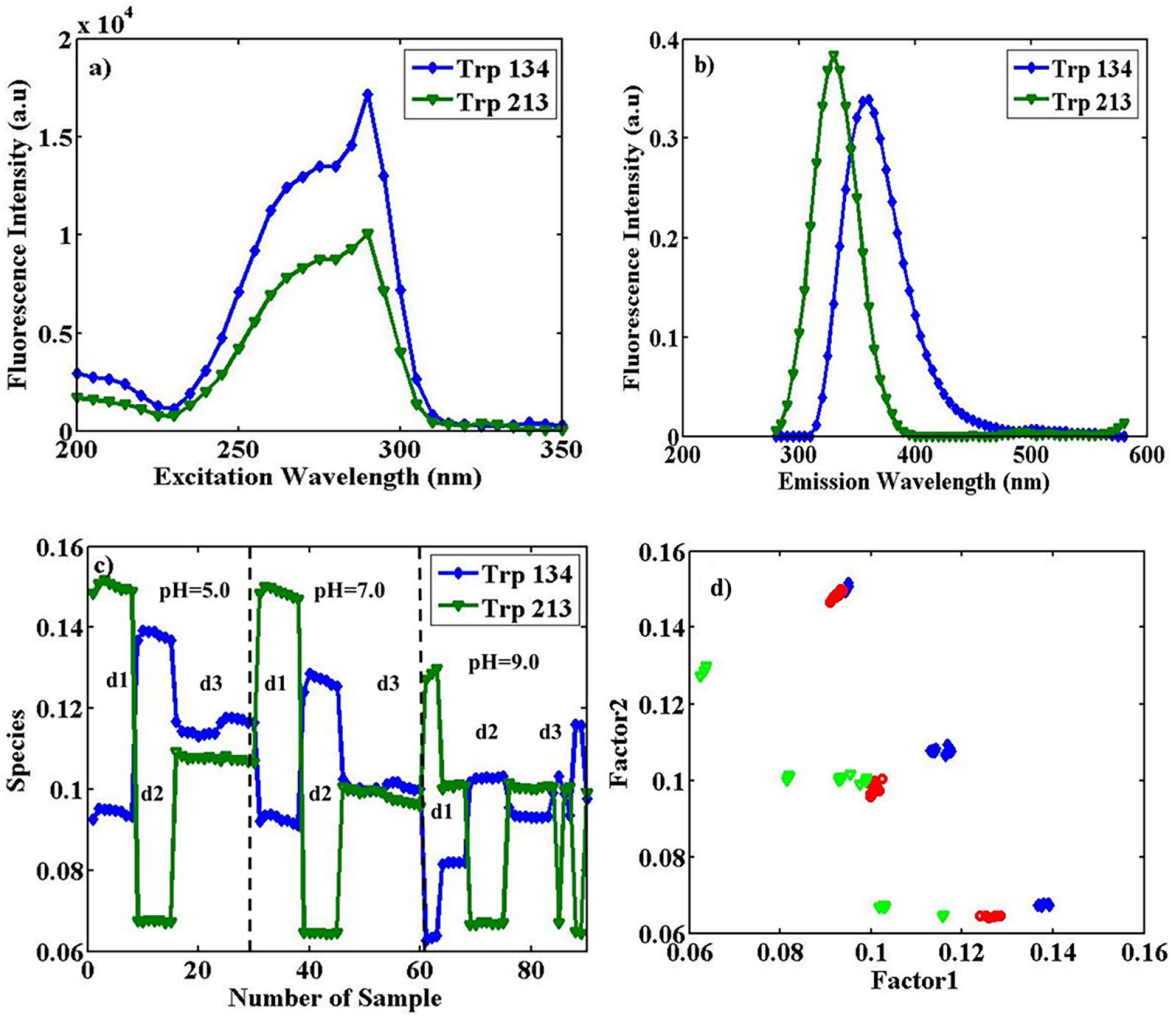
Estimated excitation spectra (**a**), emission spectra (**b**), and the contribution profiles (**c**), obtained from the application of PARAFAC with two factors on the augmented data of pure BSA with the size of 31 (λ_ex_) × 61 (λ_em_) × 90 (sample), in pHs of 5.0, 7.0 and 9.0. In the excitation profiles, with λ_ex_^max^ = 290 nm, the blue profile (lozenge shapes) is similar to Trp 134 (λ_em_^max^ = 360 nm), and the green profile (triangle shapes) Trp 213 (λ_em_^max^ = 330 nm), respectively. Thirty replicates for each pH were measured on three different days, as d1, d2, and d3, and (**d**) the loadings scatter plot in the sample (**c**) mode from the application of PARAFAC with two factors on the array with the size 31 (λ_ex_) × 61 (λ_em_) × 90 (sample) of the augmented data. The plot is factor 2 vs. factor 1; the blue lozenges are related to pure BSA samples at pH = 5.0, the red circles at pH = 7.0, and the green triangles at pH = 9.0.

*PARAFAC* As stated above, in an array with a size of 31 (λ_ex_) × 61 (λ_em_) × 90 (sample), using PARAFAC (the EEM fluorescence data sets applied in this study are trilinear) on data with two significant factors, the explained variance of 99.4%, was obtained. Therefore, two Trp types were determined. Factors 1 and 2 are related to Trp 134 (blue profile) and Trp 213 (green profile), respectively. Figure 2 indicates estimated excitation spectra, emission spectra, and contribution profiles obtained from the application of PARAFAC. As shown in Fig. 2a and b, two Trp types had approximately the same excitation profiles and various emission profiles.

The different possible conformation states of BSA take place by changing pH, so valuable information about the polarity modifications around the fluorophore molecule was obtained by shifting the emission maximum. By exposing BSA to a polar environment (solvent) with a specific protein conformation, with λ_ex_ = 290 nm, some Tryptophan is placed on the outer surface, mainly Trp134 (λ_em_^max^ = 360 nm), which is shown with blue lozenges. Green triangles show that other Trps are located in the hydrophobic environment inside the protein, frequently Trp213 (λem max = 330 nm)^36^. The discrimination between different days is more significant than between different pHs. In the acidic and natural pH, the overall fluorescence is higher for both inner and outer Trps, compared to basic pH. However, in all pH values, the replicate spectra during three different days are different. On the first day (d1) of pH = 5.0 conditions Trps are mostly inside (green profiles show higher intensities than blue ones), which is opposite to the second day (d2) and different from the third day (d3). Remarkable changes during different days at constant pH show that the conformation of BSA can be different in constant pH at different times. At the neutral pH, the sum of TrpOut and TrpIn during different days is similar to that in acidic pH conditions, and the fluorescence changes are similar. In basic conditions, the overall signals are decreased, showing lower signals of deprotonated Trps, and proteins are in a conformational condition in which fluorescence of TrpIn and TrpOut are comparable (similar to d3 of acidic and neutral pH). Therefore, the contribution profiles in Fig. 2c show a decreasing trend and represent the changes in BSA conformation and its spectral intensities on different days. Also, PARAFAC was applied on augmented data arrays using a non-negativity constraint to have a realistic solution (only positive contributions and the spectral profiles are possible).

Figure 2d includes the scatter plot of loadings in contribution mode from the employment of PARAFAC with two factors on the augmented array with dimensions 31 (λ_ex_) × 61 (λ_em_) × 90 (sample). In plot (d) in the figure, the blue lozenges are related to pure BSA samples at pH = 5.0, the red circles at pH = 7.0, and the green triangles at pH = 9.0. The plot axes are higher than zero because the nonnegativity constraint was used. Using information obtained by PARAFAC, Factor 1 and Factor 2 have the main role in discrimination between samples. As is shown in plot (2d), the pure BSA samples at mentioned pH values are almost separated along the first factor. Not properly separating BSA samples in different pH values could be due to conformational changes of proteins in solutions of the same pH values during other days. Fluorescence spectra for samples in each pH class were measured on different days. The recorded spectra on a constant pH and successive days are not in one cluster.

### Effect of temperature

*PCA* PCA with two PCs was performed on the augmented metricized data from three classes of temperature with dimensions of 90 (sample) × 1891 (λ_ex_ × λ_em_). A scatter plot of scores from the mode of BSA samples at temperatures 15.0, 25.0, and 35.0 °C resulted in no complete separation of samples in different temperatures.

*PARAFAC* An array with dimensions of 31 (λ_ex_) × 61 (λ_em_) × 90 (sample) of pure BSA fluorescence was resolved to apply PARAFAC using two significant factors with the explained variance of 99.13%, and two Trp types were specified. Factors 1 and 2 are related to Trp 134 (blue profile) and Trp 213 (green profile), respectively.

**Figure 3:**
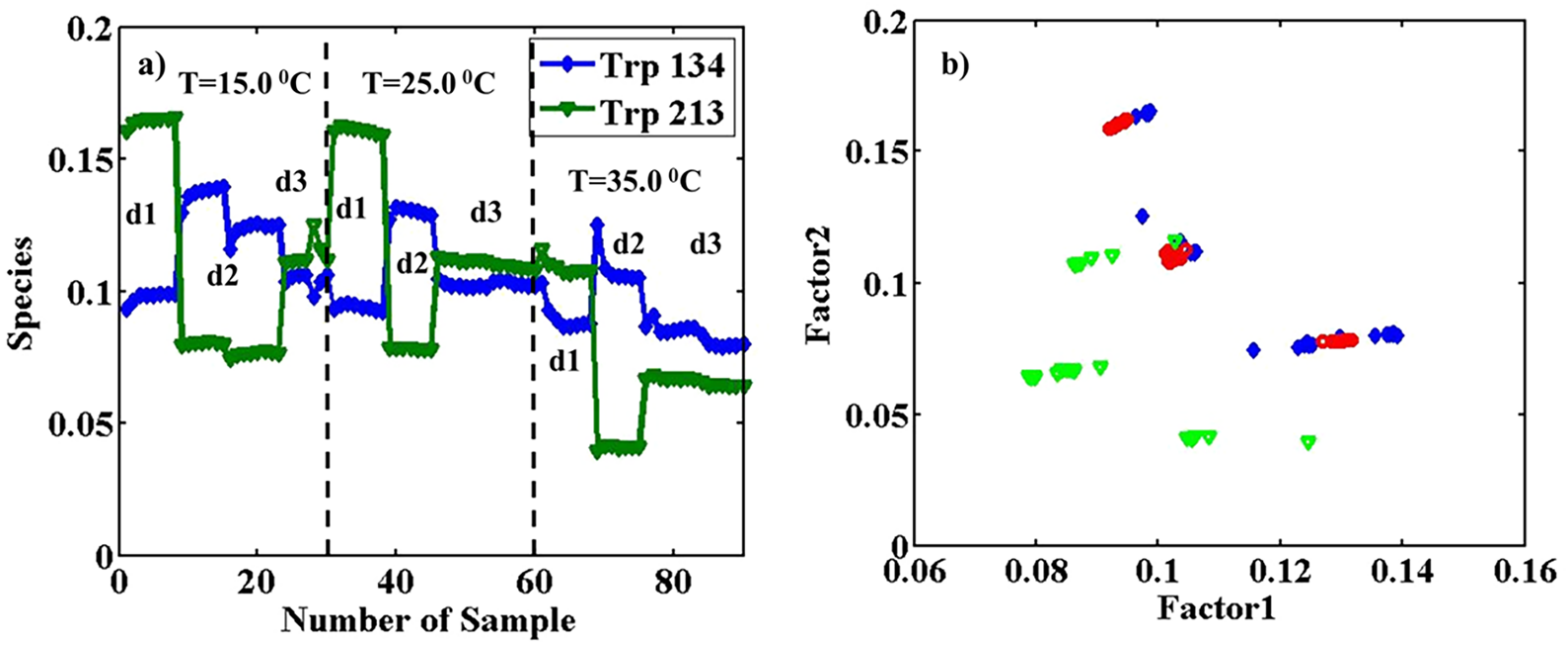
Estimated concentration profiles (**a**), obtained from the application of PARAFAC with two factors on the augmented data of pure BSA with the size of 31 (λ_ex_) × 61 (λ_em_) × 90 (sample) in the temperatures of 15.0, 25.0 and 35.0 °C. Thirty replicates for each temperature were measured on three different days, as d1, d2, and d3, and the loadings scatter plot in the contributions mode (**b**), factor 2 is plotted vs. factor 1. The blue lozenges are related to pure BSA samples at T = 15.0, the red circles at T = 25.0, and the green triangles at T = 35.0°.

Excitation and emission profiles with λ_ex_ = 290 nm for TrpOut (λ_em_^max^ = 360 nm) with blue lozenges and TrpIn (λ_em_^max^ = 330 nm) with green triangles was resolved, similar to the previous part. The different conformation states of BSA were taken place by changing temperature. In some cases, increasing temperature causes protein denaturation and permanent changes in the position of amino acids concerning their surroundings^21^. The contribution profiles in Fig. 3a show a decreasing trend with increasing temperature. These changes are less than what was observed for pH changes. Also, Fig. 3b includes the scatter plot in contributions modes from the employment of PARAFAC with two factors on the augmented array with dimensions 31 (λ_ex_) × 61 (λ_em_) × 90 (sample).

The blue lozenges are related to pure BSA samples at T = 15.0°, the red circles at T = 25.0 °C, and the green triangles at T = 35.0 °C. Figure 3b includes the replicates of pure BSA samples at mentioned temperatures and on different days and illustrates the almost complete separation of samples in T = 35 °C from that of other temperatures. However, samples from 15 °C and 25 °C are not properly separated. A more clear separation of clusters is between different days. Results show that BSA can exist in different conformations in each considered temperature with considerably different spectral behavior on various days.

## BSA/Fe samples with the same Fe concentration

### Effect of pH

*PCA* PCA with two principal components was executed on the augmented metricized data from three classes of BSA/Fe samples (with 6.0 mg Fe) at pH = 5.0, 7.0, and 9.0, with dimensions of 90 (sample) × 1891 (λ_ex_ × λ_em_). The scatter plot of PC2 vs. PC1 scores in the mode of samples was similar to Fig. 1a, and the loadings from the mode of excitation and emission wavelengths were similar to Fig. 1b. No proper separation of BSA/Fe samples in different pH values was observed in the PCs scatter plot of samples.

**Figure 4:**
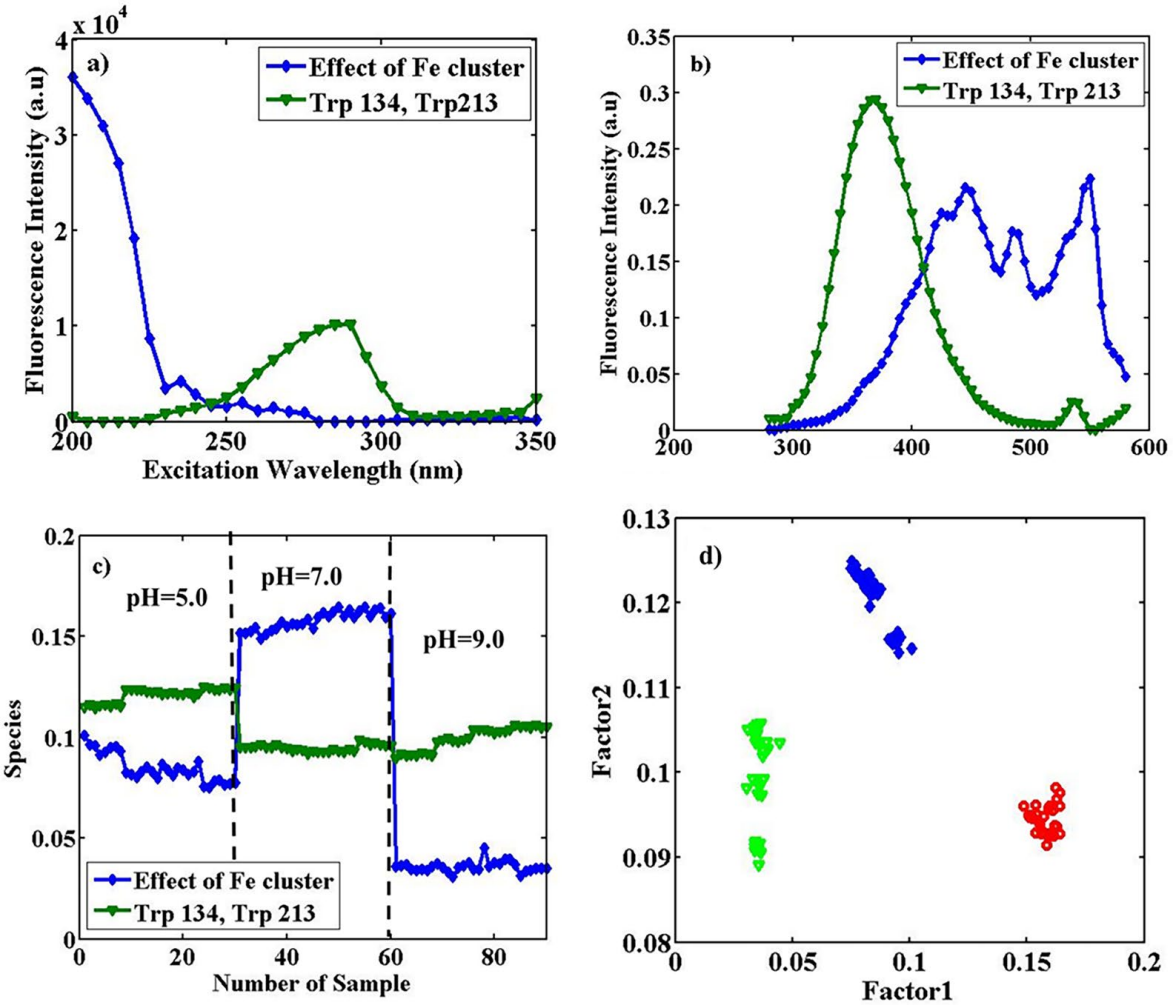
Estimated excitation spectra (**a**), emission spectra (**b**), and the contribution profiles (**c**), obtained from the application of PARAFAC with two factors on the augmented data of BSA/Fe (the concentration of Fe cluster equals 6.0 mg) with the size of 31 (λ_ex_) × 61 (λ_em_) × 90 (sample), in pHs of 5.0, 7.0 and 9.0. In the excitation profiles, with λ_ex_^max^ = 235 and 290 nm, the blue profile (lozenge shapes) is related to the effect of the Fe cluster (λ_em_^max^ = 445 nm) and the green profile (triangle shapes) Trp 134 and 213 (λ_em_^max^ = 370 nm) respectively and (**d**), the loadings scatter plot in the mode of the sample from the application of PARAFAC with two factors on the array with the size 31 (λ_ex_) × 61 (λ_em_) × 90 (sample) of the augmented data. The plot is factor 2 vs. factor 1; the blue lozenges are related to BSA/Fe samples at pH = 5.0, the red circles at pH = 7.0, and the green triangles at pH = 9.0.

*PARAFAC* The array of fluorescence data from BSA/Fe (with 6.0 mg Fe) in three different pH values mentioned above of size 31 (λ_ex_) × 61 (λ_em_) × 90 (sample) was considered. Applying PARAFAC on the data array, supposing two significant factors, led to the explained variance of 93.06%. Factor 1 shows the effect of the Fe cluster (blue profile) in solution, and Factor 2 is related to both Trp 134 and Trp 213 (green profile). Figure 4 indicates estimated excitation (a) and emission spectra (b) of the two components, in addition to contribution profiles (c) obtained from the application of PARAFAC.

The profile with λ_ex_ = 290 nm and emission with λ_em_^max^ = 370 nm (green triangles) represent both TrpIn and TrpOut, and other profiles with λ_ex_ of about 200 nm and λ_em_^max^ = 445 and 555 nm (blue lozenges) can be attributed to the presence of Fe cluster. The contribution profiles in Fig. 4c show a clear trend of changes in BSA/Fe conformation as the function of pH. Comparable intensities of profiles in this figure to that in Fig. 2 show that the Fe cluster is not a quencher for fluorescence of BSA. Figure 4c also shows the conformational (spectral) stability and reproducibility of BSA/Fe during different days. Figure 4d includes the scatter plot of loadings in contribution mode from the employment of PARAFAC with two factors on an augmented array of 31 (λ_ex_) × 61 (λ_em_) × 90 (sample) from BSA/Fe in different pH values. The blue lozenges are related to BSA/Fe samples at pH = 5.0, the red circles at pH = 7.0, and the green triangles at pH = 9.0. The BSA/Fe samples at mentioned pHs show complete separation along the first Factor. Fluorescence changes in the experiments are related to variations in protein conformation as the result of the inclusion of the Fe cluster into proteins. Changes in conformations result in changes in the environment of fluorescent amino acids in a protein. The results show the stability of BSA/Fe conformation during different days, in addition to the structure’s sensitivity to the pH of the solution. PARAFAC showed a better performance in the separation of classes in comparison to PCA.

### Effect of temperature

*PCA* PCA with two PCs was performed the same as before on data from three classes of BSA/Fe (6.0 mg Fe) at 15.0, 25.0, and 35.0 °C with dimensions of 90 (sample) × 1891 (λ_ex_ × λ_em_). A scatter plot of scores from the mode of samples shows the complete overlap between the three classes. Therefore, PCA has not been able separate the samples in the different temperatures.

**Figure 5:**
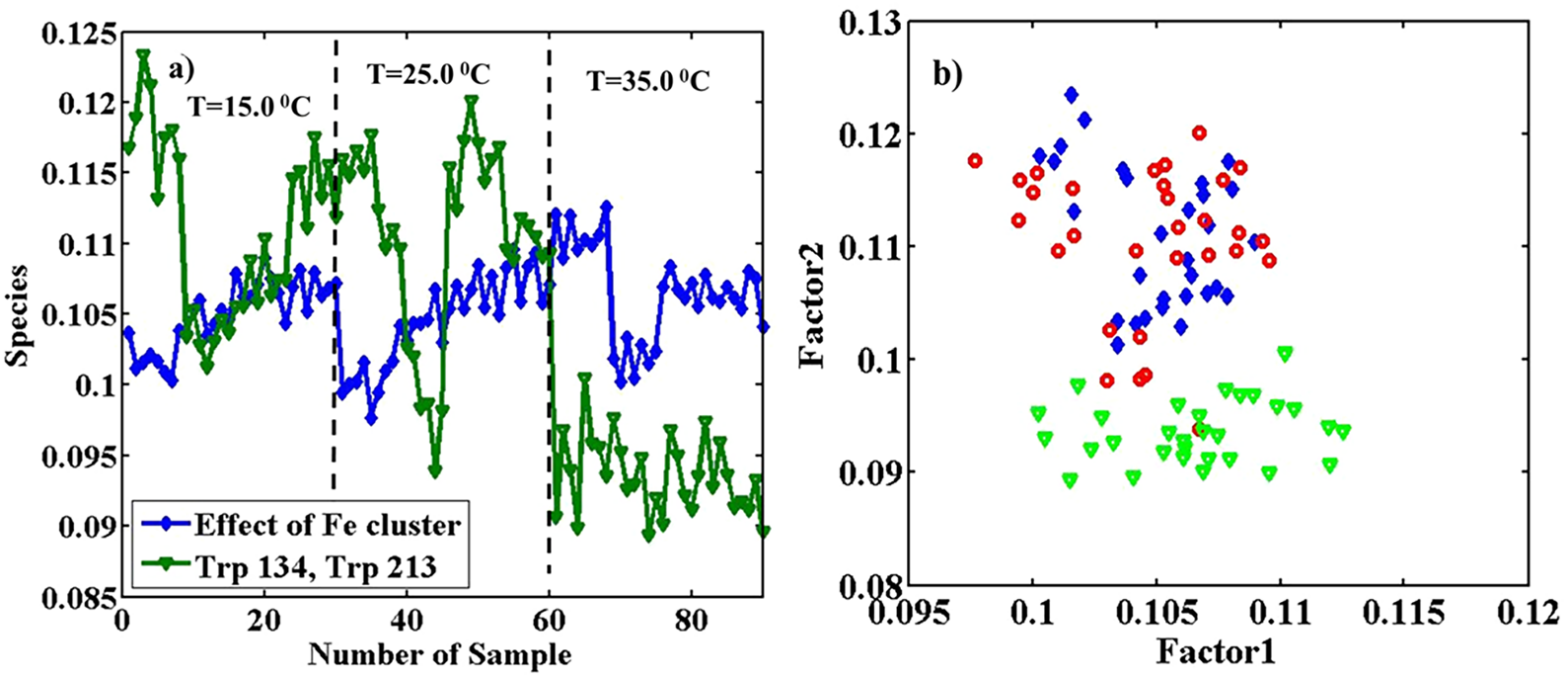
Estimated contribution profiles (**a**), obtained from the application of PARAFAC with two factors on augmented data of BSA/Fe (the concentration of Fe cluster equals 6.0 mg) with the size of 31 (λ_ex_) × 61 (λ_em_) × 90 (sample), at temperatures of 15.0, 25.0 and 35.0 °C. The blue profile (lozenge shapes) is related to the effect of the Fe cluster (λ_em_^max^ = 445 nm) and the green profile (triangle shapes) on Trp 134 and 213 (λ_em_^max^ = 370 nm), respectively, and (**b**), the loadings scatter plot in the mode of the sample from the application of PARAFAC with two factors on the array with the size 31 (λ_ex_) × 61 (λ_em_) × 90 (sample) of the augmented data. The plot is factor 2 vs. factor 1, and the blue lozenges are related to BSA/Fe samples at T = 15.0, the red circles at T = 25.0, and the green triangles at T = 35.0 °C.

*PARAFAC* Applying PARAFAC on a three-dimensional fluorescence data array of size of 31 (λ_ex_) × 61 (λ_em_) × 90 (sample), on the BSA/Fe fluorescence obtained two significant factors with the explained variance of 95.50%. Factor 1 shows the effect of the Fe cluster (blue profile) in solution and Factor 2 is related to both Trp 134 and Trp 213 (green profile). Figure 5a indicates estimated contribution profiles.

The profiles of the excitation and emission are similar to the results obtained in the pH change experiment on BSA/Fe samples (Fig. 4a,b). However, the contribution profiles trend in Fig. 5a shows the differences in the results from pH changes. It shows low spectral reproducibility on different days, which means conformation variation of proteins of the same temperature on other days. Also, Fig. 5b includes the scatter plot of loadings in contribution mode from the employment of PARAFAC. In the figure, the blue lozenges are related to BSA/Fe samples at T = 15.0, the red circles at T = 25.0, and the green triangles at T = 35.0 °C. PARAFAC can separate the samples in T = 35 °C. Thus, PARAFAC showed better results than PCA.

## BSA/Fe samples with different contributions of Fe cluster

*PCA* PCA with three PCs was applied on unfolded augmented array with size 120 (sample) × 1891 (λ_ex_ × λ_em_) from BSA sample, BSA/Fe sample containing 4.0 mg of Fe cluster, BSA/Fe sample with 6.0 mg of Fe cluster, and BSA/Fe sample including 8.0 mg of Fe cluster. A scatter plot of scores showed a trend in data points as the function of the increase in Fe cluster concentration, although no proper separation of classes was observed.

**Figure 6:**
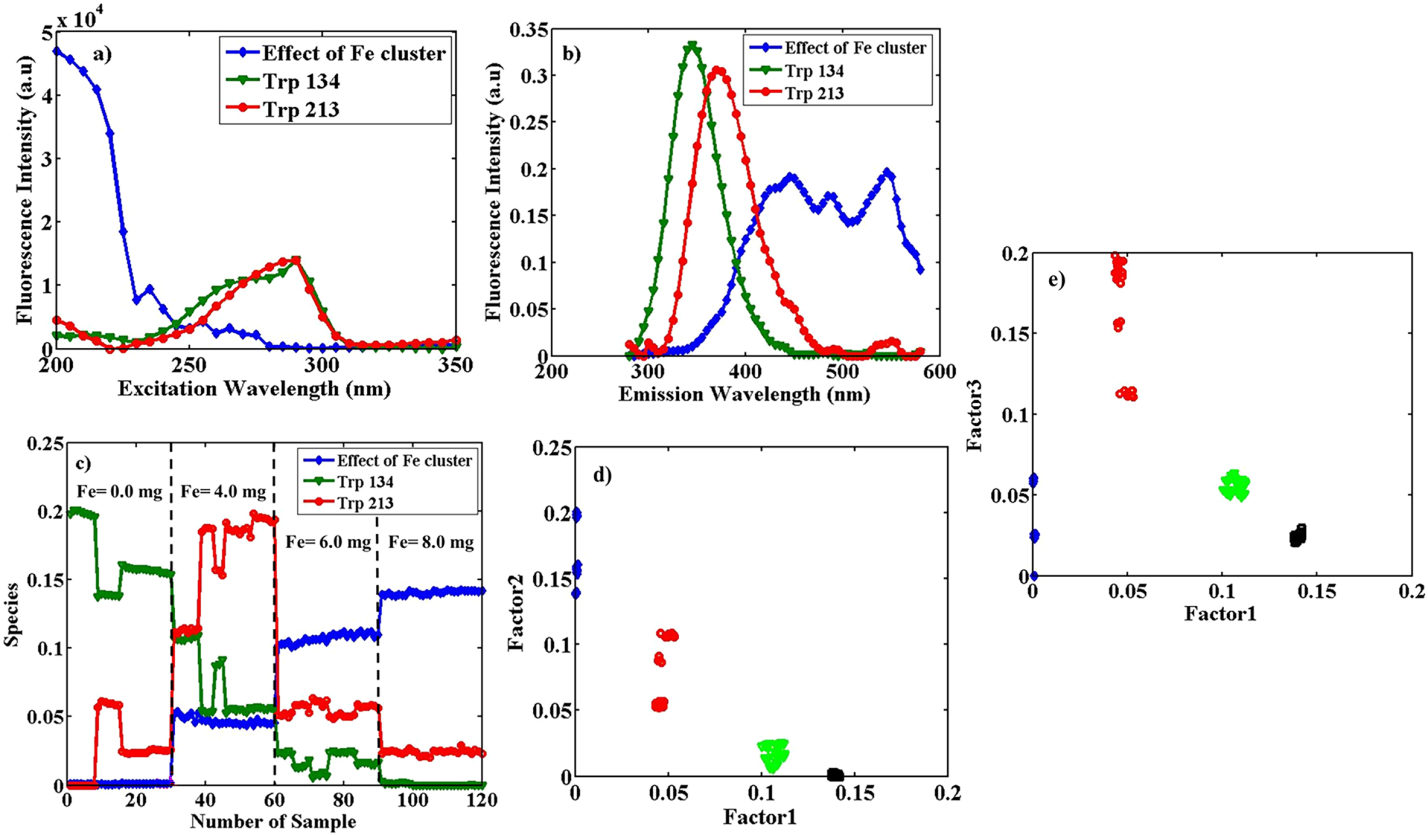
Estimated excitation spectra (**a**), emission spectra (**b**), and the contribution profiles (**c**), obtained from the application of PARAFAC with three factors on the augmented data of BSA and BSA/Fe in the different concentrations of Fe cluster of size 31 (λ_ex_) × 61 (λ_em_) × 120 (sample). In the excitation profiles, with λ_ex_^max^ = 200 nm, the blue profile (lozenge shapes) is related to the effect of the Fe cluster (λ_em_^max^ = 450 nm), and the excitation profile, with λ_ex_^max^ = 290 nm is similar to the green profile (triangle shapes) Trp 213 (λ_em_^max^ = 345 nm) and the red profile (circle shapes) Trp 134 (λ_em_^max^ = 370 nm) respectively. Also, the scores scatter plots in samples ((**d**), (factor 2 vs. factor 1) and (**e**), (factor 3 vs. factor 1)) mode from the application of PARAFAC with three factors on the array with the size 31 (λ_ex_) × 61 (λ_em_) × 120 (sample) of the augmented data. In plots (**d**) and (**e**), the blue lozenges are related to pure BSA, the red circles, the green triangles, and the black squares are related to BSA/Fe samples with the different concentrations of Fe cluster equals 4.0, 6.0 and 8.0 mg respectively.

*PARAFAC* Applying PARAFAC on the mentioned array, including fluorescence data from BSA solutions with a different concentration of Fe clusters with the dimensionality of 31 (λ_ex_) × 61 (λ_em_) × 120 (sample), three significant factors with an explained variance of 94.61% were obtained, and two Trp types were determined. Factor 1 shows the effect of the Fe cluster (blue profile) in solution, factors 2 and 3 are related to Trp 134 (green profile) and Trp 213 (red profile), respectively. Figure 6 indicates estimated excitation spectra, emission spectra, and contribution profiles obtained from PARAFAC. As shown in Fig. 6a and b, two types of component (Trp) had almost the same excitation profiles and different emission profiles.

The profiles with λ_ex_^max^ = 290 nm are due to TrpOut (λ_em_^max^ = 370 nm) and TrpIn (λ_em_^max^ = 345 nm), shown by red circles and green triangles, respectively. Also, a profile with λ_ex_ of about 200 nm and λ_em_^max^ = 450–550 nm indicates the effect of the Fe cluster on the BSA. The changes in the contribution ratio of TrpOut (often Trp134) to TrpIn (mostly Trp213) are due to conformational changes in BSA. The decrease in the contribution of both types of Trps indicates quenching due to the change in Fe cluster concentration. The contribution of TrpIn, the green profile in Fig. 6c, decreased with increasing Fe cluster inside the protein, and that of TrpOut, with the red profile, firstly increased and then decrease. Application of up to 4.0 mg Fe cluster results in a decrease of TrpIn and an increase of TrpOut (change in the ratio of contributions), which means conformational change. However, when applying 6.0 mg or higher of Fe, both TrpOut and TrpIn decrease, which means quenching of Trps fluorescence by Fe clusters.

Figure 6d and e show the scores of scatter plots in samples mode from the application of PARAFAC using three factors. The blue lozenges are related to pure BSA, the red circles, the green triangles, and the black squares are related to BSA/Fe samples with the different contributions of Fe cluster equals 4.0, 6.0, and 8.0 mg, respectively. As Fe concentration increases (from blue lozenges to black squares), values on factor 1 increase, which shows the relation of this factor to Fe concentration and its effect; the values on the Factor 2 axis decrease, which illustrates the correlation of this factor to TrpIn contribution. Values on the Factor 3 axis (Fig. 6e) first increase and then decrease with the addition of Fe. It means Factor 3 almost represents changes in TrpOut contribution.

As can be seen in Fig. 6d and e, samples with different Fe cluster concentrations are completely separated. It shows the clear effect of Fe cluster concentration on the spectral behavior of BSA. The first factor is effective for discriminating samples. Figures also show that at higher concentrations of Fe, repeatability of the measured spectra are higher and black squares (8.0 mg Fe) are pretty close to each other. However, it is not the case for red (4.0 mg Fe) and blue (6.0 mg Fe) markers. It shows that as the Fe concentration increases BSA structure becomes more rigid.

## BSA and BSA/Fe samples using different pH conditions

**Figure 7:**
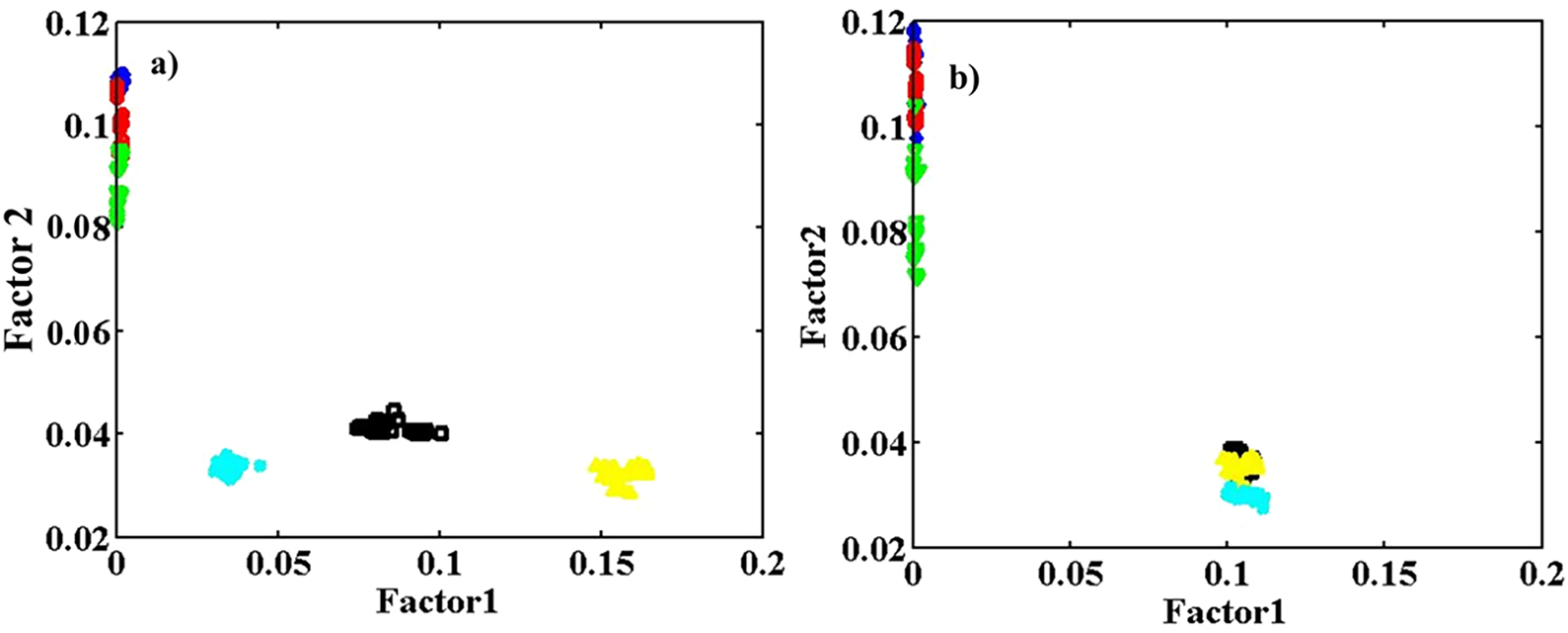
The loadings scatter plot in the sample mode (**a**), (factor 2 vs. factor 1) from the application of PARAFAC with two factors on the array with the size 31 (λ_ex_) × 61 (λ_em_) × 180 (sample) of augmented data from BSA and BSA/Fe at different pH values. The blue lozenges, the red circles, and the green triangles (upward) are related to pure BSA. Also, the black squares, the yellow triangles (downwards), and the pale blue stars are associated with BSA/Fe samples, with the concentrations of Fe cluster equal to 6.0 mg in pHs of 5.0, 7.0, and 9.0, respectively. The loadings scatter plot in the sample mode (**b**), (factor 2 vs. factor 1) from the application of PARAFAC with two factors on the array with size 31 (λ_ex_) × 61 (λ_em_) × 180 (sample) of the augmented data. Also, the blue lozenges, the red circles, and the green triangles (upward) are related to pure BSA. The black squares, the yellow triangles (downwards), and the pale blue stars are associated with BSA/Fe samples with the concentrations of Fe cluster equal to 6.0 mg in the temperatures of 15.0, 25.0, and 35.0 °C, respectively.

Applying PARAFAC, the loadings scatter plots in samples modes were calculated with two factors on the array with size 31 (λ_ex_) × 61 (λ_em_) × 180 (sample) of augmented data from BSA and BSA/Fe at three different pH values. The data from two experiments were augmented to compare the effect of pH on BSA and BSA/Fe in the same plot and in a simple visual way. Figure 7a demonstrates the plot of Factor 2 vs. Factor 1, with the blue lozenges, the red circles, and the green triangles (upward) related to pure BSA. Black squares, the yellow triangles (downwards), and the pale blue stars are associated with BSA/Fe samples with the contributions of Fe equals 6.0 mg in pHs of 5.0, 7.0, and 9.0, respectively.

The results of PARAFAC show a higher effect of pH on the conformation of Fe containing BSA than BSA. The resulting scatter plot from PCA was also examined, and it was found that PARAFAC resulted in better class separation than PCA as the result of pH change.

## BSA and BSA/Fe at different temperatures

PARAFAC was performed on the array with size 31 (λ_ex_) × 61 (λ_em_) × 180 (sample) of augmented data with two factors. The data sets of BSA and BSA/Fe in different temperatures were augmented to better compare the temperature effect on BSA and Fe-containing protein. Figure 7b shows the loadings scatter plot in sample mode. The blue lozenges, the red circles, and the green triangles (upward) are related to pure BSA. Also, the black squares, the yellow triangles (downwards), and the pale blue stars are associated with BSA/Fe samples. The contributions of Fe equals 6.0 mg in temperatures of 15.0, 25.0, and 35.0 °C, respectively.

The results of PARAFAC indicate the effect of temperature change on the samples of the pure BSA and BSA/Fe. As shown in Fig. 7b, the classes of samples with different temperatures in BSA and BSA/Fe samples are very close. Lower reproducibility of the results from BSA during different days compared to that from BSA/Fe is also obvious in the figure, which shows the lower flexibility of Fe-containing protein compared to protein without Fe cluster. It means that temperature change from 15 to 35 °C has no significant effect on protein conformations, especially on BSA/Fe. However, Fig. 7a demonstrates that applying different pH conditions causes discrimination between Fe-containing samples. Therefore, the effect of pH change on the BSA/Fe conformation is more than temperature change.

This work separated the pure BSA from BSA/Fe within different environmental conditions (pH and temperature) using low-cost fluorescence spectroscopy combined with chemometrics methods. It was found that BSA is not stable on other days, and the presence of the Fe cluster reduces this instability. The presence of different concentrations of Fe cluster was investigated, and Fe concentration was found to be effective on spectra of BSA/Fe samples. In this way, BSA with different contributions to the Fe cluster was distinguished. PCA and PARAFAC, as unsupervised classification methods, were used to obtain these observations. The results from the two methods were almost similar in some cases, but the PARAFAC as a multiway method outperformed PCA in many conditions.

Also, the extent of effect from different parameters such as pH and temperature of the solution and the presence of different concentrations of Fe cluster were examined and compared.

In the case of BSA/Fe, there were small structural changes within each class. Spectral changes in BSA proteins during different days were higher, which could be attributed to the unstable protein conformation. The pH of the solution was more effective on the spectral behavior of BSA and BSA/Fe compared to temperature, and Fe containing BSA was more sensitive to pH changes. The obtained observations in this study showed that unsupervised classification methods could be successfully applied for discriminating samples with different conformations of BSA and metal clusters containing BSA using fluorescence as a rapid, low-cost, and sensitive spectroscopic method.

## Conclusions

In this research, we have studied the conformational changes of BSA and BSA/Fe induced by pH, temperature, and different concentrations of Fe utilizing excitation-emission (EEM) fluorescence spectroscopy and unsupervised classification methods. EEM fluorescence spectroscopy as an informative technique can be coupled with PCA and PARAFAC methods for classifying and recognizing differences between protein samples. Here, better results were obtained with PARAFAC. This method has an essential advantage to PCA, that there are more interpretable estimated loadings, as they separately correspond to excitation and emission spectra of the fluorescent compounds. Fe clusters are highly effective in the pathway of denaturation of the BSA protein. The cube of spectra measured during the conformational changes was significantly different for Fe clusters containing BSA compared to pure BSA. Herein, we showed that pHs and different Fe concentrations are impressive factors in discriminating BSA from BSA/Fe samples. Therefore, a rapid, sensitive, and low-cost investigation showed the differences between these two similar proteins. Due to the continuous occurrence of conformational changes in proteins, their replicated fluorescence spectra were not as similar as those from a small molecule.

## Supplementary Information

Supplementary Information.

## Data Availability

All data generated or analyzed during this study are included in this published article [and its supplementary information files].
