# Supplementary material for: Unsupervised recognition of components from the interaction of BSA with Fe cluster in different conditions utilizing 2D fluorescence spectroscopy
Source: Sci Rep. 2022 Oct 7;12:16875. doi: 10.1038/s41598-022-20768-6 (PMC9547014; doi:10.1038/s41598-022-20768-6)
Supplement: Supplementary file 1 — Supplementary Information. [file 41598_2022_20768_MOESM1_ESM.docx]

**Supporting**

**Information**

**Unsupervised recognition of components from the interaction of BSA with Fe cluster in different conditions utilizing 2D fluorescence spectroscopy**

Mohsen Kompany-Zareh,*^a-c^ Somayyeh Akbarian ^a^, Mohammad Mahdi Najafpour ^a,b,d^

^a^Department of Chemistry, Institute for Advanced Studies in Basic Sciences (IASBS), Zanjan, 45137-66731, Iran

^b^Center of Climate Change and Global Warming, Institute for Advanced Studies in Basic Sciences (IASBS), Zanjan, 45137-66731, Iran

^c^Department of Chemistry, Dalhousie University, P.O. Box 15000, Halifax, Nova Scotia, B3H 4R2, Canada

^d^Research Center for Basic Sciences & Modern Technologies (RBST), Institute for Advanced Studies in Basic Sciences (IASBS), Zanjan 45137-66731, Iran

**Corresponding author; Phone: (+98)2433153123; Fax: (+98)2433153232; E-mail:* [*kompanym@iasbs.ac.ir*](mailto:kompanym@iasbs.ac.ir)

**Theory**

**PCA.** Principal component analysis (PCA) is one of the simplest unsupervised classification methods used for two-way data matrix decomposition into principal components. These new obtained variables that are linear combinations of the original ones and orthogonal to each other characterize the maximum sources of variance in the data.^1-3^ The first principal component is the direction that computes the most variability in the data set (maximum variance). Also, PCA can be employed on metricized three-way EEM data sets. Mathematically, the PCA model decomposes the data matrix into two sections, the bilinear terms, and a residual matrix:

$$\boldsymbol{x}_{\boldsymbol{ij}}\boldsymbol{=}\sum_{\boldsymbol{n=1}}^{\boldsymbol{N}} \boldsymbol{a}_{\boldsymbol{in}} \boldsymbol{b}_{\boldsymbol{nj}}\boldsymbol{+}\boldsymbol{e}_{\boldsymbol{ij}}\boldsymbol{i=1.\ldots.I J=1.\ldots.J (1)}$$

Where *x_ij_* is the intensity of the *i*th sample at the *j* th variable, *a_in_* is called the score value from *n* th factor, and *b_nj_* is called a loading matrix element. *e_ij_* is also an element of the residual matrix, which is the variability not computed by the model. Thus, related sections can be easily shown with data transformation by a series of scores and loading plots. Score plots indicate clustering and sample separation, while loading plots show the projection of the original variables on any principal axis.^4^

**PARAFAC.** PARAFAC is a multivariate method used for the trilinear decomposition of three-way data without rotational problems in mild conditions. For EEM data arranged in a three-way array, $\boldsymbol{X}$ *(I × J × K),* where *I*, *J*, and *K* are the number of samples, emission wavelengths, and excitation wavelengths, respectively.^5^ PARAFAC decomposes the ***X*** array into three matrices, named ***A*** (scores), ***B***, and ***C*** (loadings) with the respective elements *a_in_*, *b_jn_*, *c_kn_*, where *n* is the component number and less than the minimum of I, J, and K. An element of ***X*** is given by $\boldsymbol{x}_{\boldsymbol{ijk}}\boldsymbol{=}\sum_{\boldsymbol{n=1}}^{\boldsymbol{N}} \boldsymbol{a}_{\boldsymbol{in}} \boldsymbol{b}_{\boldsymbol{jn}}\boldsymbol{c}_{\boldsymbol{kn}}\boldsymbol{+}\boldsymbol{e}_{\boldsymbol{ijk}}\boldsymbol{(2)}$

Where *x_ijk_* is the fluorescence intensity of sample *i* at the emission wavelength *j* and excitation wavelength *k*. Also, *e_ijk_* shows an element of the residuals array **E**, including the variability not accounted for by the model.

The three-way array **X** in an n-component PARAFAC model can be expressed as follows,

$$\boldsymbol{X}=\boldsymbol{A}\left( \boldsymbol{C}ʘ\boldsymbol{B} \right)+\boldsymbol{E} (3)$$

In equation (3) **X** = [**X**_1_ **X**_2_ *. . .* **X***_K_*] is the metricized data in the first mode, and the symbol specified the Khatri-Rao product.^6^

This array is fitted to Eq (2) by least-squares to minimize the sum of the square of the residual.^5,7^ The algorithm applied to find the solution of the trilinear model is the alternating least squares (ALS),^8^ that uses loadings values in two modes for estimating parameters of the loading matrix in the third mode. This is iterated until convergence is obtained.^9^

For *n* components, the score vectors with elements directly proportional to the contributions of any sample indicate sample differences and similarities applied to look for patterns within the samples.^10,11^

**Materials and Method**

**Materials**

The BSA (Aldrich)/Fe3O (OAC)6 ClO4 (Aldrich with MW= 691.29 g.mol-1) was synthesized with this instruction which at first, 63.0 mg of BSA protein was completely dissolved in a sufficient amount of distilled water. Then each time, the desired contributions were gradually added to the Fe cluster and stirred. Then the synthesized solutions were brought to a volume of 25.0 mL with distilled water. After evaporating the water and drying the solution in the air at room temperature, the resulting brown solution was characterized by SEM, EDX, and XRD and then analyzed. The obtained images are shown in **Fig.S1** and **S2**.





**Fig.S1|** XRD patterns Fe cluster, BSA, and BSA/Fe cluster.


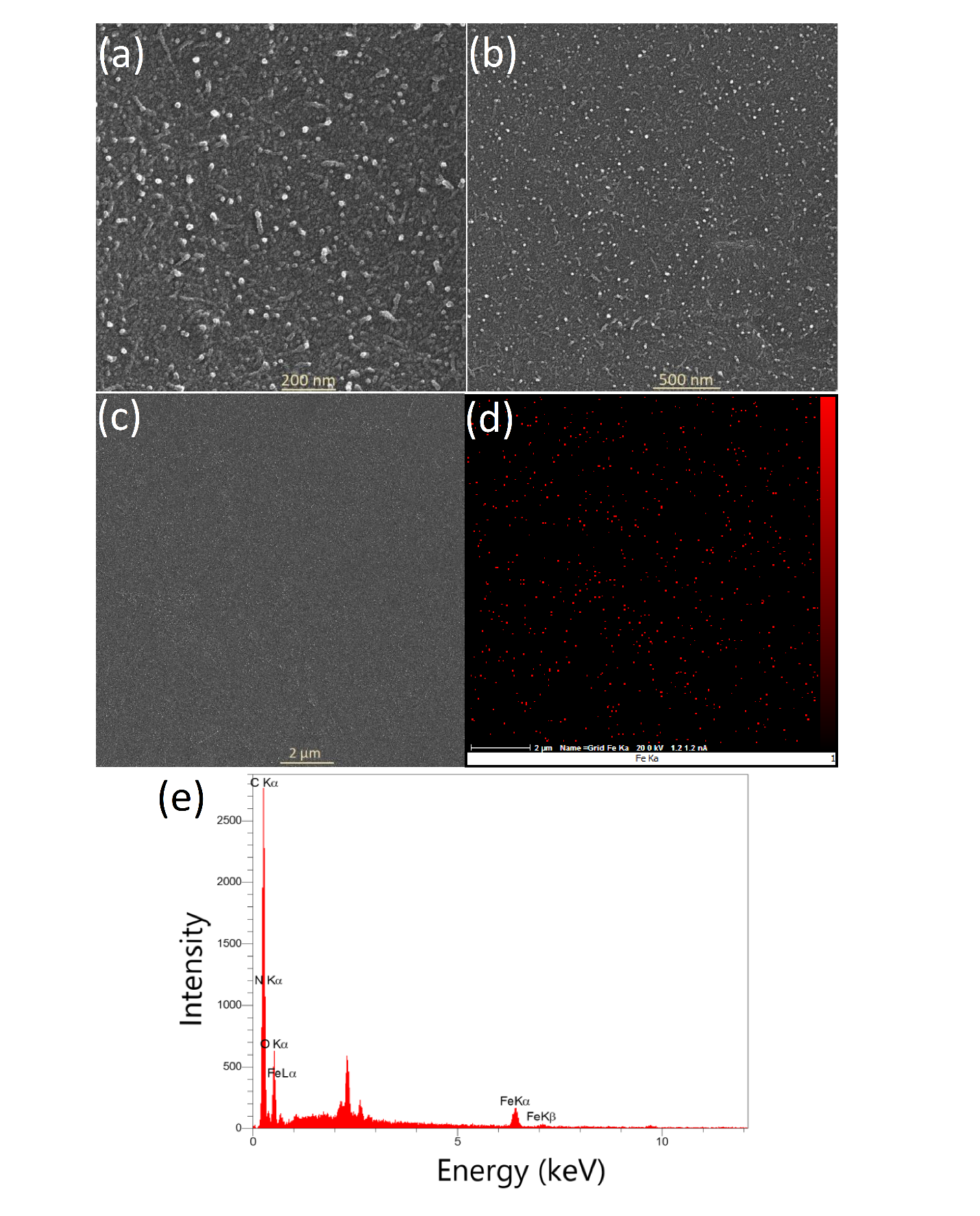


**Fig.S2|** SEM images of BSA/Fe cluster (a,b). EDX-SEM images of BSA/Fe cluster (c,d). EDX spectrum BSA/Fe cluster (e).

To investigate the synthesized compounds’ behavior, 63.0 mg of BSA protein was completely dissolved in a sufficient amount of distilled water compared to pure BSA solution. It was brought to a volume of 25.0 ml; therefore, a pure solution was prepared. Then, according to the synthesis method, BSA/Fe samples were synthesized with different contributions of Fe clusters (4.0, 6.0, and 8.0 mg). The solutions obtained by using 0.1 M hydrochloric acid (Merck) and 0.1 M sodium hydroxide (Merck) were adjusted to pH = 5.0, 7.0, and 9.0. The solutions with pH = 7.0 were used for spectroscopic examination at temperatures of 15.0, 25.0 and 35.0 0C.

**Apparatus and software**

SEM, EDX, and XRD were taken using REM JEOL JSM-7500F, VEGA\TESCAN-XMU, and Bruker, D8 ADVANCE diffractometer (Cu-Kα radiation), respectively. Also, the fluorescence spectra were measured on a Cary Eclipse Spectrofluorometer equipped with a thermostatic bath (Model of Pharmacia Biotech) using a cell with a width of 1 cm. Therefore, the ﬂuorescence excitation-emission matrix (EEM) landscapes were acquired by recording the excitation and emission spectra from 200 to 600 nm (every 5 nm). Both excitation and emission slits were set at 5 nm and the scan rate used was 9600 nm/min. All the measurements were carried out at room temperature (25 ± 0.5°C) unless stated otherwise. All chemometrics methods were written in MATLAB using version 7.12.0.635, Mathworks (R2011a), and the profiles of the PARAFAC method was obtained through PLS-Toolbox4.1.

**References**

[1] A. Gredilla, J. M. Amigo, S. F. O. De Vallejuelo, A. De Diego, R. Bro, J. M. Madariaga, *Analytical Methods* **2012**, 4, 676-684.

[2] B. Wu, J. Zhu, F. N. Najm, *IEEE Computer Society* **2004**, 660-667.

[3] M. Felipe-Sotelo, R. Tauler, I. Vives, J. O. Grimalt, *Science of the Total Environment* **2008**, 404, 148-161.

[4] K. R. Murphy, R. Bro, C. A. Stedmon, *Aquatic organic matter fluorescence* **2014**, 339-375.

[5] R. M. Callejón, J. M. Amigo, E. Pairo, S. Garmón, J. A. Ocaña, M. L. Morales, *Talanta* **2012**, 88, 456-462.

[6] Y. Akhlaghi, M. Kompany-Zareh, M. R. Hormozi-Nezhad, *Analytical Chemistry* **2012**, 84, 6603-6610.

[7] A. Muñoz De La Peña, N. Mujumdar, E. C. Heider, H. C. Goicoechea, D. Muñoz De La Peña, A. D. Campiglia, *Analytical Chemistry* **2016**, 88, 2967-2975.

[8] Y. Ni, G. Liu, S. Kokot, *Talanta* **2008**, 76, 513-521.

[9] G. Zhang, N. Zhao, L. Wang, *Journal of Luminescence* **2011**, 131, 2716-2724.

[10] I. D. Merás, J. D. Manzano, D. A. Rodríguez, A. M. de la Peña, *Talanta* **2018**,178, 751-762.

[11] L. Lenhardt, R. Bro, I. Zeković, T. Dramićanin, M. D. Dramićanin, *Food Chemistry* **2015**, 175, 284-291.
